# The Biology of *Mycobacterium Tuberculosis* Infection

**DOI:** 10.4084/MJHID.2013.070

**Published:** 2013-11-16

**Authors:** Giovanni Delogu, Michela Sali, Giovanni Fadda

**Affiliations:** 1 Istituto di Microbiologia, Università Cattolica del Sacro Cuore, Rome.

## Abstract

Tuberculosis (TB) still poses a major threat to mankind and during the last thirty years we have seen a recrudescence of the disease even in countries where TB was thought to be conquered. It is common opinion that more effective control tools such as new diagnostics, a new vaccine and new drugs are urgently needed to control the global pandemic, though the so far insufficient understanding of the *Mycobacterium tuberculosis* (Mtb) mechanism of pathogenesis is a major obstacle for the development of these control tools. In this review, we will summarize the recent advancement in the understanding of Mtb biology and on the pathogenesis of Mtb infection with emphasis on latent infection, with the change in paradigm of the last few years where the dichotomy between latent and active disease has been reconsidered in favor of a dynamic equilibrium between the host and the bacilli, encompassing a continuous spectrum of conditions that has been named TB spectrum. Implications for the diagnosis and control of disease in certain population will also be discussed.

## Introduction

Tuberculosis (TB) is one of the ancient and deadliest disease of mankind, still posing a major health, social and economic burden at a global level and primarily in low and middle income countries.^1^ The lack of an effective vaccine, the long and expensive drug regimens, the few diagnostic tools available in countries where TB is endemic and the dismantlement in several nations of the health systems and control measures that so effectively contributed to control TB throughout most of the XX century, led to the reemergence of TB as a global pandemic. The last twenty years have seen a renewed interest on TB by health authorities and governments which resulted in halving TB deaths. However, it is widely accepted that only a better understanding of the pathogenic processes associated with infection and disease will lead to the development of effective tools capable of conquering this ancient scourge. TB is one of the first and most studied infectious disease, as classically highlighted by the seminal work of R. Koch more than 100 years ago, but we have yet to answer many key questions on the mechanisms of pathogenesis and on the immunological correlates, if any, associated with protection from developing disease such as those posed by E.L. Trudeau more than a century ago.^2^

### Mycobacterium tuberculosis

#### Evolution

TB is caused by members of the specie *Mycobacterium tuberculosis* complex (MTBC), which includes: *Mycobacterium tuberculosis* (*Mtb*), the etiologic agent of TB in humans; *M. africanum*, that causes TB in humans only in certain regions of Africa; *M. bovis*, *M. caprae* and *M. pinnipedii*, causing TB in wild and domesticated mammals; *M. microti*, that causes TB only in voles. Deciphering the ≅ 4 Mbp genome provided a new understanding of the biology of the tubercle bacillus, with the identification of new and somehow unexpected properties^3^ and allowed the reconstruction of the history of *Mtb* as a global human infectious agent.^4^*Mtb* emerged as a human pathogen in Africa around 70.000 years ago and then spread out of the continent following human migrations.^5^,^6^ It is now widely accepted that the ancients *Mtb* strains originated from environmental mycobacteria (smooth tubercle bacilli),^7^ that can still be isolated from immunocompromised patients in certain parts of east Africa, are unable to cause chronic persistent infection in the immune-competent host and are not transmitted among humans. These ancient *Mtb* strains evolved, through a genetic bottleneck, so to persist in low density populations, causing disease reactivation following long period of latent infection.^8^ Following domestication, humans were able to transmit the disease to animals and *M. bovis* emerged as a pathogen of domesticated and wild animals.^4^ The introduction of agriculture, civilization and the increase in human population density in urban areas led to the selection of *Mtb* strains with enhanced virulence and transmissibility that are named modern *Mtb* strains.^9^,^10^ The modern *Mtb* strains spread throughout the world causing the TB epidemics that ravaged mankind for centuries and these strains are responsible for most of the TB cases nowadays.^11^

#### The Bacillus

*Mtb* is a slow growing mycobacteria with a doubling time of 12–24 h under optimal conditions. A major feature of *Mtb* is the peculiar cell wall structure, that provides an exceptionally strong impermeable barrier to noxious compounds and drugs and that plays a fundamental role in virulence. The classical view of the mycobacterial cell wall structure has been revised thanks to the introduction of a new electron microscopy technique, cryo-electron tomography on vitreous section, that preserves cell wall organization by avoiding sample dehydration.^12^,^13^ Thanks to these advancements it was shown that mycobacteria possess an outer membrane, functionally similar to what seen in gram-negative bacteria, consisting in an asymmetric lipid bilayer made of long fatty acids in the inner leaflet (mycolic acids) and of glycolipids and waxy components on the outer layer. The outer and inner membrane form a periplasmic space, with the presence of a thin layer of peptidoglycan in the innermost side covalently linked to arabinogalactan and lipoarabinomannan which in turn are bound to mycolic acids. Isoniazid and ethambutol, two of the most effective anti-TB drugs, target the synthesis of the mycolic acids and arabinogalactan, respectively, highlighting the importance of the mycobacterial cell wall in *Mtb* biology.

**Figure 1:**
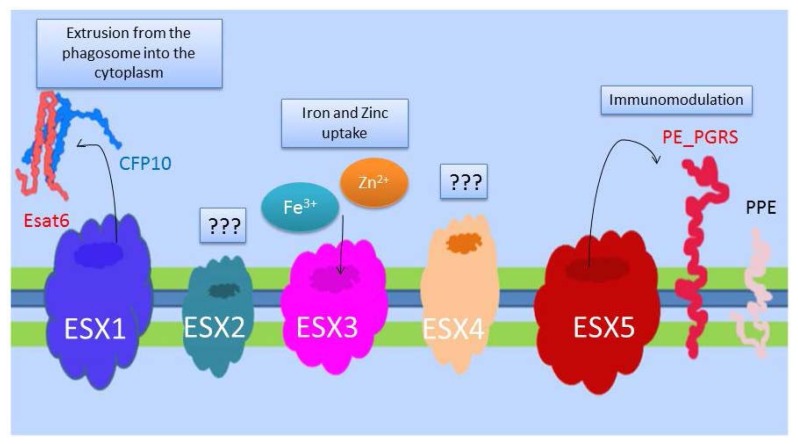
Protein Secretion systems Five different secretion system have been described in *Mtb* (titled Type VII Secretion System -T7SS), encoded by gene clusters and called ESX1 to ESX5. ESX1 and ESX5 secrete different proteins involved in the virulence of *Mtb:* ESX1 secretes antigens that interfere with the integrity of the phagosomal membrane, leading to phagosomal rupture and bacterial emission into the cytosol. ESX5 is present only in slow growing mycobacteria (such as *Mtb* and *M. marinum*) and it is thought to be involved in the secretion of proteins (PPE and PE-PGRS) with immunomodulatory properties. ESX3 is involved in Zinc and Iron uptake and homeostasis and as such is essential for growth. The role of ESX2 and ESX4 remain still unknown.

Protein secretion systems are the main virulence factors of pathogenic bacteria and in *Mtb* five type 7 secretion systems were identified (ESX1-5)(Figure 1).^14^ The best characterized of these is ESX1, which is missing in the attenuated *M. bovis* vaccine strain Bacille Calmette and Guerin.^15^,^16^ ESX1 is required for the full virulence of *Mtb*, which uses this secretion system to translocate from the phagosome into the cytosol of infected macrophages where it may persist in a protected environment.^17^–^19^ ESX1 secretes among many antigens, ESAT-6 and CFP-10, two small highly immunogenic proteins that form the basis of the immunological diagnosis of *Mtb* infection in the interferon-gamma release assays (IGRAs).^20^ Since BCG lacks ESX1 and does not express ESAT-6 and CFP-10, IGRAs can be used to detect *Mtb* infection even in subjects previously immunized with BCG, which may not be otherwise distinguished with the classical Mantoux intradermal reaction. ESX3 is involved in the acquisition of iron and zinc by *Mtb* and is essential for growth also in culture.^21^ ESX5 is found only in MTBC, *M. marinum* and *M. ulcerans* and it may represents a secretion systems specifically evolved to interact with a complex immune system such as that of mammals.^22^ While the role and function of ESX2 and ESX4 are still debated, the elucidation of the ESX systems on TB pathogenesis is certainly one of the major advancements of the last decade in the TB field, providing a new understanding of the host-pathogen interaction and very rewarding in terms of new diagnostics and potentially capable of providing new therapeutics and vaccines in the near future.

The characterization of other *Mtb* surface constituents such as the mycobacterial adhesin HBHA^23^ and PE_PGRS proteins^24^,^25^ is starting to shed light on the molecular mechanisms involved in the interaction between the bacilli and host cells, and may lead to the development of “smart” tools capable of interfering with *Mtb* pathogenesis.

Another group of proteins known to play an important role in pathogenesis are those under the control of the dormancy survival regulon (Dos), which controls expression of more than 50 genes responsible for the *Mtb* hypoxic response.^26^,^27^*Mtb* senses the harsh environment in macrophages and granulomas, characterized by low oxygen and nutrient depletion, and responds by activating a dormant state, whereby the bacilli stops multiplying, down-regulate central metabolism and activate anaerobic metabolism, with induction of stress proteins that provide *Mtb* with unique biological and immunological features.^28^ These metabolically active but not replicating dormant bacilli can persist for a long time *in vivo* and may revert to an active state thanks to the resuscitating promoting factors (rpf), which act on the peptidoglycan to trigger a cascade of events that promotes bacterial growth.^29^,^30^ Hence, *Mtb* persists in host tissues under different metabolic states, with important implications from a pathogenetic and clinical practical perspectives, since dormant bacteria are susceptible only to certain antibiotics (pyrazinamide, rifampin and metronidazole) but resistant to other such as isoniazid.^31^

## TB Pathogenesis

**Figure 2:**
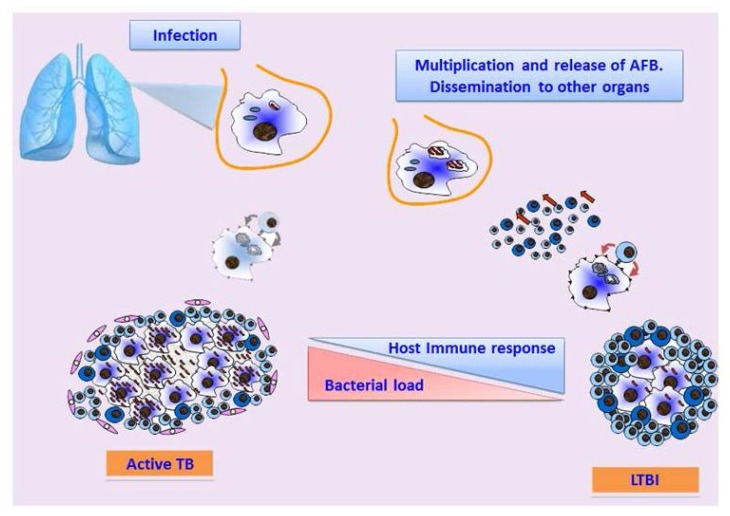
TB pathogenesis Tubercle bacilli are inhaled in aerosol droplets, enter into the lungs and, when the host innate immune defenses fail to eliminate the bacteria, *Mtb* start multiplying inside alveolar macrophages and then spreads to other tissues and organs through the bloodstream and lymphatics. Once the cell-mediated immune response kicks in, bacterial replication is usually controlled and in 90–95% of cases non overt signs or symptoms of disease ensue (Latent TB). During latent infection a dynamic equilibrium between the bacilli and host immune responses is established and any event that weakens cell mediated immunity may lead to active bacterial replication, tissue damage and disease occurs (active TB).

*Mtb* infection occurs when few tubercle bacilli dispersed in the air from a patient with active pulmonary TB reach the alveoli of the host. Here, *Mtb* is quickly phagocytized by professional alveolar macrophages that most often can kill the entering bacteria thanks to the innate immune response (Figure 2).^32^ If the bacilli can survive this first line of defense, it starts actively replicating in macrophages, diffuse to nearby cells including epithelial and endothelial cells, reaching in few weeks of exponential growth a high bacterial burden.^33^ During these early steps of infection, *Mtb* can diffuse to other organs through the lymphatics and by haematogenous dissemination where it can infect other cells.^34^ Thereafter, once the adaptive immune response kicks in, migration to the site of primary infection of neutrophils, lymphocytes and other immune cells form a cellular infiltrate that later assume the typical structure of a granuloma.^35^ Fibrotic components cover the granuloma that becomes calcified such that bacilli remain encapsulated inside and protected by the host immune response. This primary lesion, classically termed the Ghon complex,^36^ was thought to be the “sanctuary” of *Mtb* during latent infection, with bacilli persisting in a dormant, non-metabolically active state, for years, decades, or most often for lifetime. In this scenario, when, during latent infection, for unknown reasons, bacilli would start replicating inside this primary lesion, active disease would ensue.^37^ A major corollary of this hypothesis, with relevant pathophysiological and clinical implications, was that reactivation of TB originated from this very primary site of infection. This hypothesis was challenged since the early 20^th^ century, when it was shown that viable and infective bacilli were found in unaffected portion of lung tissues of infected guinea pigs or human necropsy rather than from the central core of the tuberculous lesions.^37^,^38^ Despite these early findings, only in 2000 Hernandez-Pando et al,^39^ using normal lung tissues isolated at necropsy from patients who had died for causes other than TB in a TB endemic country, were able to detect by in situ PCR *Mtb* DNA in non-phagocytic cells, fibroblasts and endothelial cells, clearly suggesting that in latent TB subjects *Mtb* bacilli can persists in tissues and cells not associated with the granuloma or the Ghon complex. Using similar experimental settings, *Mtb* was detected in the fat tissue surrounding several organs, residing intracellularly in adipocytes, where it can survive protected from the host immune response.^40^ All these evidences suggest that during LTBI *Mtb* can reside in different organs, tissues and cell types, not associated with the site of primary infection and lacking any sign of the typical granulomatous lesions.

Studies carried out in the non-human primate model of TB further corroborated these findings indicating that during latent infection *Mtb* is metabolically active and replicates in host tissues despite the lack of any clinical sign or symptom of disease.^41^,^42^ Interestingly, in a single monkey with active TB it was possible to observe many different type of lesions, ranging from liquefied cavities with massive loads of bacilli, to necrotic or caseous hypoxic lesions with variable number of bacteria, to sterile lesions.^43^ A similar scenario was observed in patients with pulmonary TB, where diverse lesions were observed simultaneously and with lesions responding differently to chemotherapy,^43^ suggesting that they represent distinct *Mtb* subsets in different microenvironments.

Based on the new understanding of the biology of *Mtb*, its different metabolic states, the dynamic host immune responses occurring during infection and on the spectrum of conditions that are observed during infection, it has been proposed that during latent infection most bacilli persist in a dormant state with fewer *Mtb* found in an active replicating state. These replicating bacilli, named “scouts” are processed and killed by the host immune defenses and as a result they are responsible for the induction of the large number of effector/memory T cells directed against *Mtb* antigens that are found in the peripheral blood.^44^ Hence, during latent TB dormant bacteria constantly replenish the bulk of actively replicating bacilli readily killed by the host. When, for any reason, host immune responses fail to control these scouts, uncontrolled bacterial replication promotes diseases manifestations and active disease ensues.^45^ Classical examples are highlighted by HIV infection that affects CD4 T cells that play a pivotal role in controlling *Mtb* replication;^46^ treatment with biological therapies with anti-TNF that are known to increase the risk of developing TB disease up to 25 times in latent TB subjects as a result of the disruption of granuloma organization and depletion of certain populations of CD8 T cells known to play a role in controlling *Mtb*;^47^,^48^ treatment with corticosteroids, vitamin D deficiency and any other condition affecting T cell function are also known to increase the risk of active TB in latent TB subjects, underscoring the clinical implications that any event capable of perturbing the host-pathogen dynamic equilibrium can have.

Cancer patients, including those with haematological diseases, are also at increased risk of developing TB and in these patients clinical outcomes are usually very aggressive, may present as systemic infections with a high fatality rate and diagnosis is usually delayed.^49^,^50^ The context of the TB spectrum, with the immunological and biological implications previously discussed, clearly highlight the risk that an infection usually controlled by the host immune response with no clinical signs or symptoms, can reactivate once the subtle balance affecting the dynamic equilibrium between the host and the bacilli occurs. Hence, it is very important to deploy proper and effective diagnostic protocols capable of detecting latent infection in these high risk groups and very sensitive assays to identify active disease when TB is suspected.

## TB Diagnosis

### Direct diagnosis

Definitive diagnosis of TB requires the detection of *Mtb* from the biological sample by at least one of the current microbiological techniques: microscopical analysis, isolation in culture or molecular methods. These assays form the basis for the microbiological diagnosis of TB and the clinicians may require detection of *Mtb* in one or more specimens depending on the clinical symptoms, if any.^51^ High sensitivity and specificity has been observed in the detection of *Mtb* in specimens such as sputum, bronchoalveolar lavage or induced sputum for the diagnosis of pulmonary TB.^52^ The introduction of new, highly sensitive, fully automated molecular assays for the detection of *Mtb* has been recognized as a major achievement of the last decades,^53^ though it is important to remind that molecular diagnosis should not be ordered routinely when the clinical suspicion of TB is too low.^54^–^56^ Non-pulmonary forms of TB may be more problematic to diagnose because of the difficulties in identifying the proper specimens and the lower sensitivity of the microbiological assays in the non-pulmonary specimens, probably resulting from a lower bacterial concentration. Detection of *Mtb* in the urine or stools could be used to detect systemic infections and recently new assays capable of detecting mycobacterial components (lipoarabinomannan, LAM) in the urine were shown to be helpful to diagnose TB in HIV-infected subjects and immunocompromised patients, but not to diagnose pulmonary TB in immunocompetent subjects.^57^,^58^

Detection of *Mtb* in clinical specimens has been observed in HIV patients not showing any clinical sign or symptom of the disease^59^ and in a recent report on the diagnosis of TB in children it was shown that ≈25% of children positive for *Mtb* did not show any clinical sign or symptom.^60^ These results highlight the challenges associated with TB diagnosis and provide clinical evidences for the TB spectrum concepts.^43^

## Immunological Diagnosis

The immunological diagnosis of TB has been historically performed by the Mantoux test or tuberculin skin test (TST) and the introduction in the last decade of the interferon-gamma release assays (IGRAs), that measure T cell responses directed against *Mtb* specific antigens in peripheral whole blood, has provided a new and valuable tool in the diagnosis of *Mtb* infection. Discussion of the immunological diagnosis of TB is beyond the scope of this review, but it is worth mentioning that TST and IGRAs are aimed at detecting *Mtb* infection but cannot distinguish between LTBI subjects with non signs or symptoms of disease and active TB patients.^61^,^62^ Despite many efforts, the prognostic value of IGRAs was shown to be insufficient and while many experimental assays are being devised and tested with the attempt to improve the current RD1-based assays,^63^ it is important to remind that IGRAs can be used only as an “aid” in the diagnosis of TB and cannot be used alone to rule out TB nor to make conclusive diagnosis of TB. The concept of the TB spectrum discussed in this review provides the biological and immunological framework to support this statement.
